# Engineering Characteristics of Cement Composites Containing a Chitosan-Based Polymer and Steel Slag Aggregates

**DOI:** 10.3390/polym14030626

**Published:** 2022-02-06

**Authors:** Se-Jin Choi, Sung-Ho Bae, Hoe Young Choi, Haye Min Ko

**Affiliations:** 1 Department of Architectural Engineering, Wonkwang University, 460 Iksan-daero, Iksan 54538, Korea; csj2378@wku.ac.kr (S.-J.C.); caos1344@naver.com (S.-H.B.); 2 Department of Chemistry, Wonkwang University, 460 Iksan-daero, Iksan 54538, Korea; wonderlost1412@naver.com; 3 Department of Chemistry & Wonkwang, Institute of Material Science and Technology, Wonkwang University, 460 Iksan-daero, Iksan 54538, Korea

**Keywords:** cement composite, steel slag aggregate, chitosan-based polymer, compressive strength, chloride-ion penetration resistance

## Abstract

Recently, sustainable development has attracted significant global attention. Toward this, several studies have been performed on the development of alternative aggregates for mortar or concrete to prevent environmental damage and rapid depletion of natural aggregates. In this study, we investigated the applicability of a chitosan-based polymer (CBP), a biomimetic polymer, to cement mortar using steel slag as a fine aggregate. The CBP was synthesized via an amide coupling reaction among chitosan, 1-ethyl-3-(3-dimethylaminopropyl)carbodiimide hydrochloride, and 3-(3,4-dihydroxyphenyl)propionic acid. Upon addition to cement mortar using natural sand or a blast furnace slag aggregate, the CBP contributed toward increasing the compressive strength and tensile strength. However, in mortar mixes using a ferronickel slag aggregate, the tensile strength decreased by ~5.7–25.4% upon CBP addition. Moreover, the CBP reduced the total charge passed through the mixes. In particular, in the mortar mix using the steel slag aggregate, the CBP showed improved chloride-ion penetration resistance. The results showed that the as-prepared CBP was a suitable improving agent and exhibited promising compatibility with cement composites containing steel slag aggregates.

## 1. Introduction

In the concrete industry, several studies have been conducted toward the development of alternatives to natural aggregates, as well as the use of industrial by-products as aggregates, to prevent environmental damage and the depletion of natural aggregates [1,2,3,4]. Steel slag, a by-product of the steel industry, is generated in the form of an aggregate, and has a particle size similar to that of natural aggregates. Therefore, it is more suitable for application in mortar or concrete compared to other industrial by-products [5,6,7]. Recently, in addition to blast furnace slag (BS), which is a representative slag aggregate, ferronickel slag (FS), which is generated as a by-product in the nickel industry, has attracted significant attention as an aggregate for mortar or concrete [8,9,10,11,12,13,14,15]. Saha et al. [11] revealed that the compressive strength of mortar with a mixture of FS and natural sand (NS) as the aggregate increased when ≤50% FS was used. Liu et al. [12] investigated the durability of concrete using a FS fine aggregate and found that the sulfate resistance of concrete improved when 27% FS was used. Ngii et al. [13] used a mixture of FS and NS as the aggregate and found that an FS content of 25% was optimum to increase the compressive strength of concrete. However, despite these efforts, the recycling rate of FS is not high [16].

Polymer materials have been widely used to improve the performance of cement mortar or concrete [17,18,19,20,21,22,23,24]. Douba et al. [20] investigated the properties of highly ductile polymer concrete and found that the samples using carbon nanotubes and polymer materials showed improved ductility. Niaki et al. [21] studied the mechanical and thermal properties of epoxy-based polymer concrete using basalt fibers and nanoclay and confirmed that the addition of basalt fibers improved the mechanical properties and thermal stability of polymer concrete. Wang et al. [22] added scrap tire rubber, an industrial by-product, to epoxy polymer concrete, and found that the compression and tensile strengths of concrete improved when 5% solid rubber was added. In addition, Asdollah et al. [23] reported that the fracture toughness of polymer concrete can be improved by adding polyethylene terephthalate (PET) filler materials (prepared by crushing recycled PET bottles). However, most previous studies that used polymer materials in mortar or concrete focused on natural aggregates; there has been no report on cement composites using steel slag aggregate and biomimetic polymers. Hence, in this study, we investigated the applicability of a chitosan-based polymer (CBP), a biomimetic polymer, to cement mortar using steel slag as a fine aggregate.

Chitin, which is extracted from marine shells, is one of the most prevalent natural polysaccharides and is considered as the best material to produce chitosan through a deacetylation process. Generally, chitin is used for the production of chitosan owing to its low cost, and because the chemical reactions involved in chitosan production are suitable for mass production [24]. This chemical process involves the treatment of chitin with sodium hydroxide at >80 °C [25]. Thus, chitosan is obtained as a random copolymer with D-glucosamine and *N*-acetyl-D-glucosamine units linked by β-1,4 glycosidic linkages. The degree of deacetylation is determined by the mass ratio of the D-glucosamine and *N*-acetyl-D-glucosamine units [26,27], and affects the molecular weight of the polymer.

Recently, chitosan has attracted significant attention owing to its bioactivity, biodegradability, biocompatibility, and wide applicability in food, pharmaceutical, textile, and tissue engineering industries. However, chitosan is soluble only in aqueous acidic media [28], which limits its further applications. Therefore, to expand the application scope of chitosan, phenolic acid groups such as catechol are grafted onto its backbone using various amide coupling reactions to improve its solubility [29,30]. Phenolic acid grafting improves not only the solubility but also the physicochemical properties of chitosan. Additionally, chitosan containing phenolic acids has shown biological activities (antioxidant, antimicrobial, anti-allergic, or anticancer) superior to those exhibited by pure chitosan. Motivated by these findings, a few researchers have investigated the use of chitosan for improving the properties of cement mortar. The compressive strength and carbonation resistance of cement mortar can be improved by adding optimal amounts of biomimetic polymers to it [31]. Therefore, in this study, we synthesized a CBP and added it to cement mortar containing steel slag aggregate. In addition, we evaluated the compressive strength, tensile strength, accelerated carbonation depth, and chloride-ion penetrability of the resulting cement mortar.

## 2. Materials and Methods

### 2.1. Materials

Chitosan (medium molecular weight; degree of deacetylation: 75–85%) and hydrocaffeic acid (HCA; 3-(3,4-dihydroxyphenyl)propionic acid)) were purchased from Sigma-Aldrich. 1-Ethyl-3-(3-dimethylaminopropyl)carbodiimide hydrochloride (EDC) was purchased from TCI; ethanol was purchased from Fisher Scientific. All chemicals were of analytical grade and were used without further purification. A dialysis membrane (Spectra/Por MWCO: 12–14 kDa) was purchased from Spectrum Laboratories, Inc. Ultrapure water was obtained by distillation using a Q-Grad 1 purification cartridge from Millipore Water Purification Systems.

**Figure 1:**
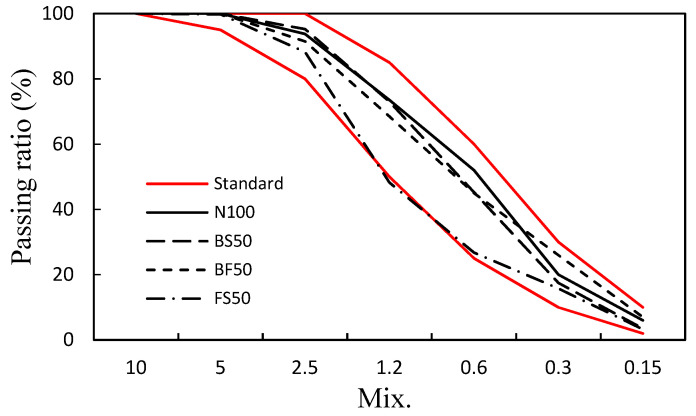
Particle size distribution of the cement mortar mixes.

In addition, ordinary Portland cement (Asia Cement Co. Ltd., Seoul, Korea) with a specific gravity of 3.15 g/cm^3^ and Blaine of 3430 cm^2^/g was used. NS (sand from the Namwon region, Korea) was used as a natural fine aggregate. BS and FS supplied by POSCO, Korea, were used as the steel slag fine aggregates. NS, BS, and FS had specific gravities of 2.60, 2.81, and 3.05 g/cm^3^, respectively, with fineness moduli (FM) of 2.89, 2.37, and 3.51, respectively, as shown in Table 1. In this study, considering the standard particle size distribution of the fine aggregates, the maximum amount of steel slag used was fixed at 50%. The following samples were prepared: N100, cement mortar with 100% NS as the fine aggregate; BS50, cement mortar wherein 50% NS was replaced with BS (by volume); BF50, wherein 25% NS was replaced with BS and another 25% was replaced with FS; and FS50, wherein 50% NS was replaced with FS. The particle size distribution curve of each aggregate was consistent with the standard particle size range, as shown in Figure 1.

### 2.2. Mix Proportions and Specimen Preparation

Table 2 lists the mix proportions of the cement mortar mixes. The water–cement ratio was fixed at 50%, and a CBP solution containing 500 mg of the CBP dissolved in 1000 mL of water was added to the cement mortar mixes. The amount of the CBP solution added with respect to the water content of the cement mortar was 0 or 10.0%.

Cubic specimens with dimensions of 50 mm × 50 mm × 50 mm were prepared via molding for compressive strength testing, and cylindrical specimens with dimensions of 50 mm × 100 mm were prepared for split-tensile strength testing. Additionally, for accelerated carbonation and chloride-ion penetration tests, specimens with dimensions of 40 mm × 40 mm × 160 mm and 100 mm × 50 mm, respectively, were prepared. Each specimen was demolded after 24 h and cured in water at 20 °C. The flow and compressive strength of the cement mortar mixes were measured according to the KS L 5105 standard [32], and the tensile strength was measured according to the KS F 2423 standard [33]. In the accelerated carbonation test, the carbonation depth was measured using a phenolphthalein solution after carbonation in an accelerated carbonation chamber according to the KS F 2584 standard [34]. In addition, the chloride-ion penetration test was performed according to the ASTM C1202 standard [35]. Scanning electron microscopy (SEM) images of the cement mortar mixes were recorded using an AIS1800C scanning electron microscope (SERON Technologies, Anseong, Korea).

### 2.3. CBP Synthesis

The CBP was synthesized according to a previously reported procedure [36,37]. First, chitosan (500 mg) was dissolved in distilled water (DW, 50 mL), and the pH of the solution was adjusted to 5.5 by adding 1.0 M HCl. A solution of HCA (500 mg) in ethanol/DW (10 mL/10 mL) and EDC (500 mg) in ethanol/DW (2.5 mL/2.5 mL) was added dropwise to the chitosan solution. The pH of the reaction mixture was maintained at 5.5 by adding 1.0 M HCl, and the reaction mixture was stirred at room temperature for 12 h. The mixture was dialyzed in a 0.1 M NaCl solution (NaCl 30 g/4.5 L DW), and the pH of the mixture was adjusted to 5.0 by adding 1.0 M HCl for 2 days, followed by the addition of 4.5 L DW for 4 h. The residue was frozen at −20 °C in a refrigerator and then lyophilized.

The absorbance of the aqueous CBP solution was measured using an ultraviolet-visible (UV-Vis) spectrophotometer (GENESYS 180, Thermo Fisher, Waltham, MA, USA). To obtain a proton nuclear magnetic resonance (^1^H-NMR) spectrum (500 MHz, JEOL) of the CBP sample, it was dissolved in D_2_O. A Fourier-transform infrared (FT-IR, Nicolet iS5, Thermo Fisher Scientific) spectrum of the CBP sample was recorded over the wavenumber range of 400–4000 cm^−1^ at a resolution of 1 cm^−1^.

## 3. Results and Discussion

### 3.1. CBP Characteristics

**Figure 2:**
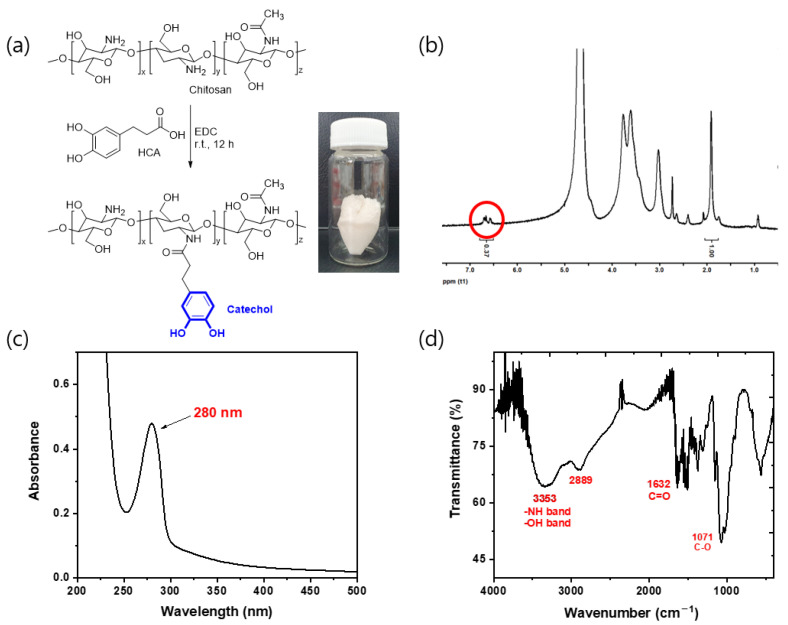
(**a**) Synthesis of the CBP. (**b**) ^1^H-NMR, (**c**) UV-Vis, and (**d**) FT-IR spectra of the CBP.

The CBP was synthesized via an amide coupling reaction between chitosan, EDC, and HCA (Figure 2a). After synthesis, a white-sponge-like solid was obtained. The structure of the CBP sample was analyzed through ^1^H NMR, UV-Vis, and FT-IR spectroscopies. The catechol group in the chitosan backbone played a crucial role in enhancing the solubility of the CBP in water by decreasing the strength of intramolecular hydrogen bond interactions. The relative ratio of the protons between the catechol and acetyl groups was determined through ^1^H NMR spectroscopy to calculate the degree of catechol conjugation (DOC_cat_) in the CBP, which was found to be ~7% (Figure 2b). In addition, the DOC_cat_ in the CBP was determined from its UV-Vis absorption peak at 280 nm using the absorption peak of HCA as the reference (Figure 2c). The results indicated that ~4% of the amino groups in chitosan reacted with HCA to form amides conjugated with 3,4-dihydroxyhydrocinnamic acid groups. Furthermore, the FT-IR spectrum of the CBP showed peaks at ~3353 and 1632 cm^−1^ corresponding to the hydroxyl and amine groups and the carbonyl groups of the amides, respectively (Figure 2d).

### 3.2. Mortar Flow

**Figure 3:**
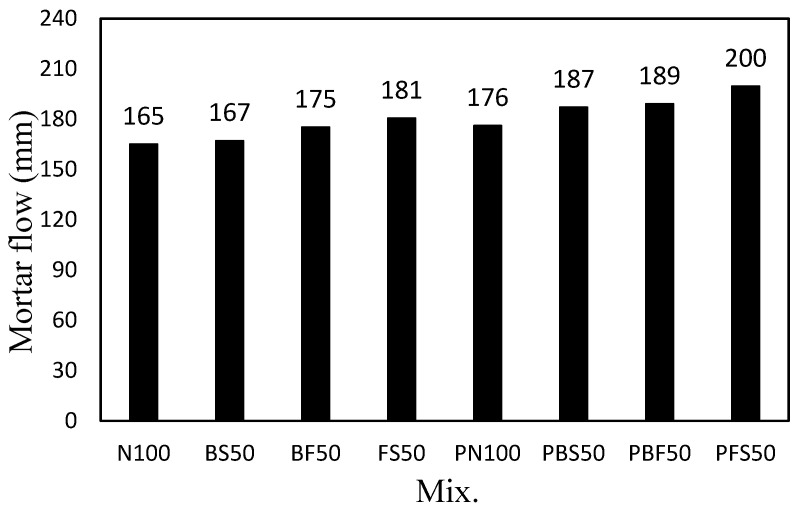
Mortar flow of the cement mortar mixes.

Figure 3 shows the flow of the mortar mixes containing the steel slag aggregate and the CBP. N100, which comprised only NS as the aggregate, showed the lowest flow of ~165 mm. BS50 (with 50% BS) showed a flow of ~167 mm, which is comparable to that of N100. The flow of FS50 (with 50% FS) was ~185 mm, which is 12.1% higher than that of N100. The mortar flow increased with the addition of the CBP, regardless of the fine aggregate used. The flow of PN100 (with CBP and NS) was ~176 mm, which is ~6.6% higher than that of N100. The flow of the mixes using the CBP and steel slag aggregates was ~187–200 mm, which was ~10.4–11.9% higher than that of the mixes without the CBP. The results indicate that the addition of the CBP led to more effective improvement in the fluidity of the mortar mixes using the steel slag aggregates than that of the mortar using only the natural aggregate.

### 3.3. Compressive Strength

**Figure 4:**
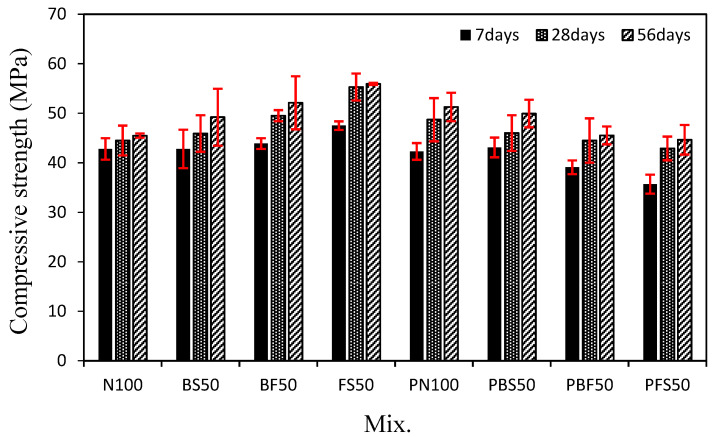
Compressive strength of the cement mortars.

Figure 4 shows the change in the compressive strength of the mortar mixes using the steel slag aggregates and CBP with time. After 7 days, the compressive strengths of N100 and BS50 were similar (~42.8 MPa), and that of FS50 was ~47.5 MPa, which is ~10.9% higher than that of N100. This increase in strength can be attributed to the high density and low absorption of FS [38]. The compressive strengths of N100 and BS50 were similar to those of PN100 and PBS50, respectively. However, the 7-day compressive strengths of PBF50 and PFS50 were lower than those of BF50 and FS50, respectively.

Even after 28 days, N100 and BS50 showed similar compressive strengths (~45.0 MPa). The 28-day compressive strengths of BF50 and FS50 were ~49.5 and 55.3 MPa, respectively, which are ~11.2% and 24.2% higher than that of N100, respectively.

**Figure 5:**
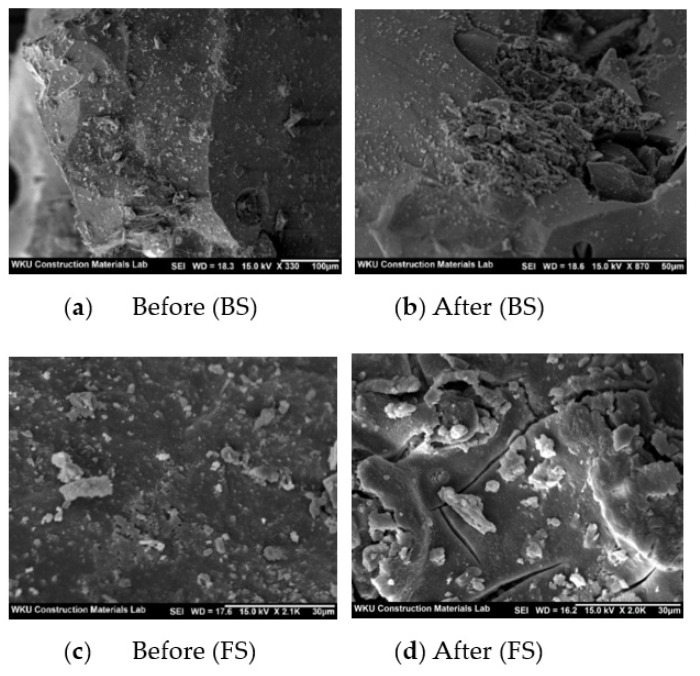
SEM images of the BS and FS samples after immersion in the CBP solution.

However, the compressive strength of the mixes using the CBP showed a completely different trend. The 28-day compressive strength of PN100 was ~48.7 MPa, which is ~9.4% higher than that of N100. The use of biomimetic polymers is effective in enhancing the compressive strength of mortar [31]. The compressive strength of PBS50 was ~46.0 MPa, which is similar to that of BS50 (45.9 MPa). In contrast, the 28-day compressive strengths of PBF50 and PFS50 were ~44.5 and 42.9 MPa, respectively, which are lower than those of BF50 and FS50 (~10.1% and 22.4%, respectively). The lower compressive strengths of PBF50 and PFS50 can be attributed to the weak compatibility between the CBP and FS. Figure 5 shows the SEM images of the BS and FS samples before and after immersion in the CBP solution for 7 days. The surface of the BS sample remains unchanged after immersion. However, numerous cracks are observed on the surface of the FS sample after immersion. Therefore, BS was found to be more suitable (as a steel slag aggregate) than FS for improving the compressive strength of the cement mortar mixes with the CBP.

The 56-day compressive strength of the mortar mixes showed a trend similar to that of the 28-day compressive strength. FS50 showed the highest 56-day compressive strength of ~55.9 MPa. However, PBF50 and PFS50 showed 56-day compressive strengths of ~44.7 and 45.5 MPa, respectively, which are ~12.6% and 20.0% lower than those of BF50 and FS50, respectively. In addition, PN100 and PBS50 showed 56-day compressive strengths of 51.3 and 49.9 MPa, respectively, which are higher than those of N100 (45.5 MPa) and BS50 (49.2 MPa).

### 3.4. Split-Tensile Strength

**Figure 6:**
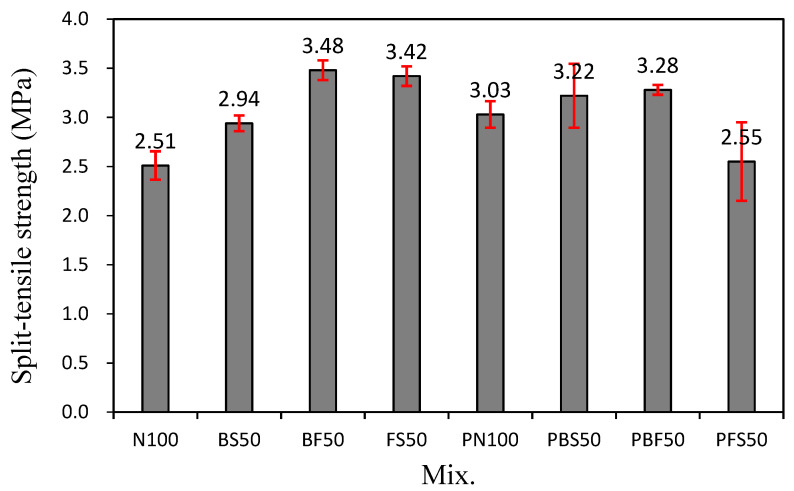
Split-tensile strength of the cement mortars.

Figure 6 shows the split-tensile strength of the cement mortar mixes after 28 days of curing. The tensile strength of N100 was ~2.51 MPa. The tensile strength of PN100 was ~3.03 MPa, which is ~20.7% higher than that of N100. In addition, the 28-day tensile strength of PBS50 (3.22 MPa) was slightly higher than that of BS50 (2.94 MPa). Therefore, for cement mortar mixes using NS or BS, the addition of the CBP resulted in an increase in the compressive and tensile strengths.

However, the tensile strengths of BF50 and FS50 were ~3.48 and 3.42 MPa, respectively. In contrast, the tensile strengths of PBF50 and PFS50 were ~3.28 and 2.55 MPa, respectively. Thus, when BF or FS was used as the fine aggregate, the tensile strength of the mixes with the CBP was ~5.7–25.4% lower than that without the CBP.

### 3.5. Carbonation Depth

**Figure 7:**
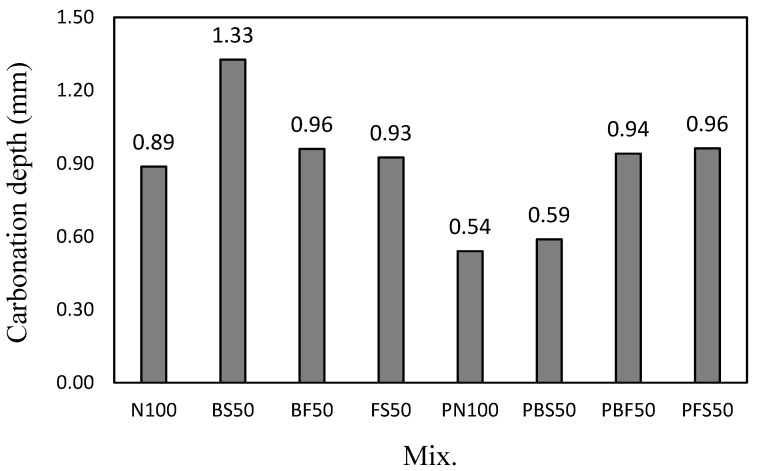
Carbonation depth of the cement mortars.

Figure 7 shows the carbonation depth of the cement mortar mixes after 28 days of accelerated carbonation. The carbonation depth of N100 was ~0.89 mm. The carbonation depth of PN100 was ~0.54 mm, which is ~39.3% lower than that of N100. The lower carbonation depth of PN100 can be attributed to its higher compressive strength than that of N100.

**Figure 8:**
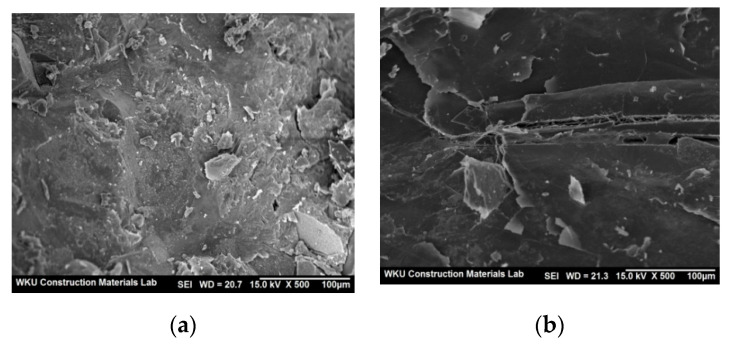
SEM images of BS50 and PBS50 after 56 days. (**a**) BS50, (**b**) PBS50.

BS50 showed the largest carbonation depth of ~1.33 mm. The carbonation depths of BF50 and FS50 were ~0.96 and 0.93 mm, respectively, which are lower than that of BS50. However, although PBS50 showed a 56-day compressive strength similar to that of BS50, its carbonation depth (~0.59 mm) was remarkably lower (55.6%) than that of BS50. This is because the product formed by the reaction between the CBP with BS contributed toward making the cement-hardened matrix denser. Figure 8 shows the SEM images of BS50 and PBS50. The surface of PBS50 is relatively denser than that of BS50. Considering that the compressive strengths of the two mixes (BS50 and PBS50) were high (≥49 MPa), it can be stated that an increase in the hardness of the cement-hardened matrix did not affect the strength of the cement mortar mixes, but effectively prevented the penetration of the carbon dioxide gas.

### 3.6. Chloride-ion Penetrability

**Figure 9:**
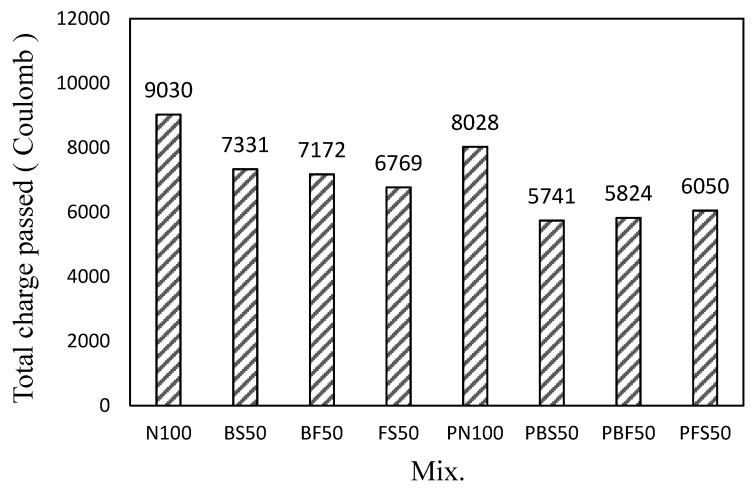
Chloride-ion penetrability of the cement mortars.

Figure 9 shows the total charge passed through the cement mortar mixes using the steel slag aggregate and CBP after 28 days. The total charge passed through N100 was the highest (~9030 C), which is 11.0% higher than that through PN100 (~8028 C). The total amounts of charge passed through BS50, BF50, and FS50 were 7331, 7172, and 6769 C, respectively, which are lower than that of N100 (~18.8–25.3%). Thus, the total charge passed through FS50 was lower than that passed through the cement mortars. The total charge passed through the mixes using the steel slag aggregates and CBP was ~5741–6050 C, which is 24.6–28.4% lower than that of PN100. The total charge passed through the mixes decreased after the addition of the CBP. In particular, the chloride-ion penetration resistance of the mortar mixes increased when the steel slag aggregates and CBP were used.

## 4. Conclusions

(1) The 56-day compressive strengths of PN100 and PBS50 were higher than those of N100 and BS50, respectively. However, the compressive strengths of PBF50 and PFS50 were lower than those of BF50 and FS50, respectively. Therefore, BS was more effective than FS for improving the compressive strength of the mixes wherein the CBP and steel slag aggregates were used.
(2) In the samples using NS or BS, the compressive and tensile strengths improved when the CBP was added. However, in the samples using FS, the tensile strength decreased by ~5.7–25.4% after CBP addition.
(3) The carbonation depth of PBS50 was ~0.59 mm, which is remarkably (~55.6%) lower than that of BS50.
(4) The total charge passed through the samples decreased after CBP addition. The chloride-ion penetration resistance of the mortar samples increased when both CBP and steel slag aggregates were used.

The findings of this study confirm that the as-prepared CBP is a promising material for improving the properties of cement mortar, and is highly compatible with cement composites containing BS aggregates.

## Figures and Tables

**Table 1:** Physical properties of the fine aggregates used in this study.

Fine Aggregates	FM	Density (g/cm^3^)	Water Absorption Ratio (%)
NS	2.89	2.60	1.0
BS	2.37	2.81	2.1
FS	3.51	3.05	0.6

**Table 2:** Mix proportions of the cement mortar mixes.

Mix	CBP Solution (wt%)	Fine Aggregate (%)			W/C (%)	Cement (kg/m^3^)	Water (kg/m^3^)
		NS	BS	FS			
N100	0	100	–	–			
BS50	0	50	50	–			
BF50	0	50	25	25			
FS50	0	50	–	50	50	340	170
PN100	10	100	–	–			
PBS50	10	50	50				
PBF50	10	50	25	25			
PFS50	10	50	–	50			

## Data Availability

Not applicable.
